# Etravirine Pharmacokinetics in HIV-Infected Pregnant Women

**DOI:** 10.3389/fphar.2016.00239

**Published:** 2016-08-04

**Authors:** Nikki Mulligan, Stein Schalkwijk, Brookie M. Best, Angela Colbers, Jiajia Wang, Edmund V. Capparelli, José Moltó, Alice M. Stek, Graham Taylor, Elizabeth Smith, Carmen Hidalgo Tenorio, Nahida Chakhtoura, Marjo van Kasteren, Courtney V. Fletcher, Mark Mirochnick, David Burger

**Affiliations:** ^1^Skaggs School of Pharmacy and Pharmaceutical Sciences, University of CaliforniaSan Diego, La Jolla, CA, USA; ^2^Department of Pharmacy, Radboud University Medical CenterNijmegen, Netherlands; ^3^Center for Biostatistics in AIDS Research, Harvard School of Public HealthBoston, MA, USA; ^4^Fundació Lluita contra la Sida, Hospital Universitari Germans Trias I PujolBadalona, Spain; ^5^Maternal Child and Adolescent/Adult Center, University of Southern California School of MedicineLos Angeles, CA, USA; ^6^Imperial College Healthcare National Health Service TrustLondon, UK; ^7^Maternal, Adolescent, and Pediatric Research Branch, National Institute of Allergy and Infectious DiseasesBethesda, MD, USA; ^8^Hospital Universitario Virgen de las Nieves GranadaGranada, Spain; ^9^Maternal and Pediatric Infectious Disease Branch, Eunice Kennedy Shriver National Institute of Child Health and Human DevelopmentBethesda, MD, USA; ^10^Department of Internal Medicine, St. Elisabeth HospitalTilburg, Netherlands; ^11^Antiviral Pharmacology Laboratory, College of Pharmacy, University of Nebraska Medical CenterOmaha, NE, USA; ^12^Department of Pediatrics, Boston University School of MedicineBoston, MA, USA

**Keywords:** etravirine, pregnancy, HIV, pharmacokinetics, perinatal transmission

## Abstract

**Background:** The study goal was to describe etravirine pharmacokinetics during pregnancy and postpartum in HIV-infected women.

**Methods:** IMPAACT P1026s and PANNA are on-going, non-randomized, open-label, parallel-group, multi-center phase-IV prospective studies in HIV-infected pregnant women. Intensive steady-state 12-h pharmacokinetic profiles were performed from 2nd trimester through postpartum. Etravirine was measured at two labs using validated ultra performance liquid chromatography (detection limits: 0.020 and 0.026 mcg/mL).

**Results:** Fifteen women took etravirine 200 mg twice-daily. Etravirine AUC_0–12_ was higher in the 3rd trimester compared to paired postpartum data by 34% (median 8.3 vs. 5.3 mcg*h/mL, *p* = 0.068). Etravirine apparent oral clearance was significantly lower in the 3rd trimester of pregnancy compared to paired postpartum data by 52% (median 24 vs. 38 L/h, *p* = 0.025). The median ratio of cord blood to maternal plasma concentration at delivery was 0.52 (range: 0.19–4.25) and no perinatal transmission occurred.

**Conclusion:** Etravirine apparent oral clearance is reduced and exposure increased during the third trimester of pregnancy. Based on prior dose-ranging and safety data, no dose adjustment is necessary for maternal health but the effects of etravirine *in utero* are unknown. Maternal health and infant outcomes should be closely monitored until further infant safety data are available.

**Clinical Trial registration:** The IMPAACT protocol P1026s and PANNA study are registered at ClinicalTrials.gov under NCT00042289 and NCT00825929.

## Introduction

In 2013, 16 million women were living with human immunodeficiency virus (HIV) infection, 1.3–1.6 million of these women were pregnant and perinatal transmission accounted for an estimated 90% of newly HIV-infected children (Prevention of mother-to-child transmission, 2015). The use of combination antiretroviral therapy (ART) can reduce the risk of perinatal transmission from ≥20 to <1% while also protecting the health and survival of the mother (HIV among pregnant women, infants, and children in the United States, 2012). Current National Institute of Health (NIH) guidelines at aidsinfo.nih.gov and the World Health Organization (WHO) recommend combination ART for all pregnant women regardless of CD4 count (Panel on Treatment of HIV-Infected Pregnant Women and Prevention of Perinatal Transmission, 2014; Prevention of mother-to-child transmission, 2015). Combination ART coverage for pregnant women in low to middle income countries has been steadily rising from 47% in 2009, to 56% in 2011, and 67% in 2013 (HIV/AIDS data and statistics, 2013).

Appropriate drug therapy in pregnancy is complicated by the many physiological changes that impact drug absorption, distribution, metabolism, and excretion. These changes include increases in gastrointestinal transit time, gastric pH, total body water, and fat which impact drug absorption and distribution. Increases in several hormones can induce metabolic pathways, compete for protein binding and compete for metabolic enzymes impacting free drug concentration and metabolism. Excretion is affected by increased hepatic plasma flow, renal plasma flow, and increased glomerular filtration rate. Further impacts to drug disposition occur due to decreases in gastric acid secretions, decreases in plasma proteins via dilution, nausea, and vomiting (Mirochnick and Capparelli, 2004). Pregnant women are a largely unstudied population in clinical trials and many antiretrovirals are initially recommended at standard adult doses due to limited or absent pharmacokinetic data during pregnancy.

Subtherapeutic antiretroviral exposure during pregnancy can lead to increased perinatal transmission, development of drug resistant mutations, and disease progression while elevations in antiretroviral exposure can lead to toxicity. Antiretroviral exposure across the placenta is crucial to the fetus, with the potential to provide prophylaxis against HIV infection or cause fetal toxicity (Mirochnick et al., 2010). Previous pharmacokinetic studies have typically shown reduced exposure of antiretroviral drugs during pregnancy with the greatest reductions seen with boosted protease inhibitors. Among first generation non-nucleoside reverse transcriptase inhibitors (NNRTIs) chronically administered throughout pregnancy, efavirenz plasma concentrations are reduced and nevirapine plasma concentrations are not significantly different during pregnancy. Etravirine and rilpivirine, second generation NNRTIs, lack sufficient pharmacokinetic data in pregnancy (Colbers et al., 2014; Panel on Treatment of HIV-Infected Pregnant Women and Prevention of Perinatal Transmission, 2014).

Etravirine is a second generation NNRTI that was approved by the US Food and Drug Administration (FDA) and the European Medicines Agency (EMA) in 2008 for use in treatment-experienced patients with human immunodeficiency virus type 1 (HIV-1). Etravirine is less susceptible to resistance with activity against 55 of 65 HIV-1 strains with single point mutation resistance to other NNRTIs (Product information, 2014). As such, etravirine holds an important place in the treatment of resistant HIV-1 strains. The recommended dose of etravirine in non-pregnant adults is 200 mg twice-daily. Etravirine is 99.9% protein bound and is a substrate for CYP2C19, CYP2C9, and CYP3A4 (Product information, 2014). CYP3A4 and CYP2C9 activity increases during pregnancy while CYP2C19 activity is inhibited (Anderson, 2005; Ke et al., 2014). To date, etravirine studies in pregnancy have been limited to a small phase IIIb clinical trial and several case studies (Furco et al., 2009; Jaworsky et al., 2010; Izurieta et al., 2011; Calcagno et al., 2013; Shust et al., 2014; Ramgopal et al., 2016).

The primary objective of this study was to describe etravirine pharmacokinetics in HIV-infected pregnant women receiving standard adult doses and to compare 2nd and 3rd trimester etravirine exposure to postpartum. A secondary objective of this study was to describe transplacental passage of etravirine with cord blood to maternal plasma ratios at delivery. This study represents a collaboration between The International Maternal Pediatric and Adolescent AIDS Clinical Trials (IMPAACT) Network (P1026s protocol) and the “Pharmacokinetics of Newly Developed Antiretroviral Agents in HIV-infected Pregnant Women” (PANNA) Network.

## Methods

### Study population and design

The IMPAACT protocol P1026s, “Pharmacokinetic Properties of Antiretroviral Drugs during Pregnancy,” enrolled subjects from sites in the Americas and is registered at ClinicalTrials.gov under NCT00042289. The PANNA study enrolled subjects from sites in Europe and is registered under NCT00825929. Both studies are ongoing non-randomized, open-label, parallel-group, multi-center phase-IV prospective studies in HIV-infected pregnant women.

These studies recruited pregnant HIV-infected women receiving etravirine prescribed as part of clinical care. Maternal inclusion criteria were pregnancy, two-sample confirmation of HIV-infection status, use of etravirine 200 mg twice-daily, 2-week stability on a combination ART regimen, and intention to continue ART treatment through 6–12 weeks postpartum. Maternal exclusion criteria were multiple gestations, a clinical or laboratory toxicity that would likely require changing medication during the study period, and the use of specific medications known to interfere with etravirine disposition. Subjects were enrolled as early as the 2nd or 3rd trimester with the expectation to complete all subsequent sampling time points.

Each study site received ethical and local institutional review board approval. All subjects gave informed consent prior to study participation. Medications were not furnished through this study but were provided by each subject's clinical care providers by prescription. Pharmacokinetic sampling was performed during the second trimester if possible, third trimester, and postpartum. In P1026s, samples were assayed in real time and results reported to each study participant and her clinician. Participant and clinician were also notified if etravirine pharmacokinetic exposure was subtherapeutic, set at the 10th percentile AUC_0–12_ for non-pregnant adults, to make informed dose adjustments if deemed necessary by the clinical care provider.

### Clinical and laboratory monitoring

Each site visit for maternal plasma sampling included monitoring of HIV-1 RNA, CD4+ lymphocyte cell count, hematology, and serum biochemistry. The lower limit of detection for HIV-1 RNA assays performed locally ranged from 20 to 400 copies/mL. A physical exam, history of concomitant medications and perinatal adherence questionnaire were also collected at each visit. All infants received physical examinations after birth while laboratory evaluations were only performed if clinically indicated. Clinical and laboratory toxicities were assessed through medical histories, physical examinations and laboratory testing on each pharmacokinetic sampling day and at delivery. Adverse events were reported at each study visit and toxicity management was determined by each participant's clinician.

### Sample collection

Samples were collected over 12 h for pharmacokinetic evaluation in the second trimester (20–28 weeks gestation), third trimester (30–38 weeks gestation), and between 3 and 12 weeks following delivery as maternal physiological conditions generally normalize to non-pregnant adult conditions within 6 weeks postpartum. Cardiac output, heart rate, and stroke volume normalize within 24–72 h postpartum while respiratory parameters normalize within 6–12 weeks postpartum to non-pregnant adult levels (Datta et al., 2010). CYP1A2 enzymes appear to normalize to non-pregnant adult levels of activity by 4 weeks postpartum. The time-course of normalization following pregnancy is unknown for other CYP450 enzymes (Anderson, 2005). Requirements included etravirine dosing after meals, adherence for 2 weeks prior to pharmacokinetic sampling and consistent dosing times for the 3 days prior to pharmacokinetic sampling. On sampling days the pre-dose sample was drawn and etravirine was administered under observation at a time consistent with previous doses. Serial blood collection was drawn by intravenous catheter from an arm vein at pre-dose, 1, 2, 4, 6, 8, and 12 h post-dose with additional time points at 0.5 and 3 h for PANNA subjects. At delivery, one maternal plasma sample and one umbilical cord blood sample were drawn after cord clamping.

### Etravirine concentration assays

Etravirine concentration assays for IMPAACT samples were analyzed at the Antiviral Pharmacology Laboratory at the University of Nebraska Medical Center by validated reversed-phase ultra performance liquid chromatography (UPLC). The lower limit of quantification was 0.020 mcg/mL. The linear fit detection ranges in plasma were from 0.020 to 20 mcg/mL. Validation results displayed a greatest mean inter-assay percent deviation of 2.6% with corresponding precision (within day, coefficient of variation) of 3.5%. The greatest mean intra-assay percent deviation was −15% and <3.8% at the lower limit of quantitation (LLOQ) and above the LLOQ, respectively, with a corresponding precision value (within day, coefficient of variation) of 3.9%. Concentration assays for PANNA samples were analyzed at the Radboud university medical center, Nijmegen, The Netherlands by UPLC. The lower limit of quantification was 0.026 mcg/mL. The linear calibration ranges in plasma were from 0.026 to 15.78 mcg/mL. Validation results displayed an accuracy of the Quality Control samples of 105, 104, and 100% at the plasma concentrations 0.37, 1.48, and 9.25 mcg/mL with corresponding precision values (within day, coefficient of variation) of 2.0, 2.3, and 0.3%, respectively. Both laboratories adhere to Clinical Laboratory Improvement Amendments (CLIA) and perform standardized interlaboratory testing though the AIDS Clinical Trial Group (ACTG) clinical pharmacology quality assurance and quality control program.

### Pharmacokinetic analyses

Etravirine maximum, minimum, and last plasma concentrations (C_max_, C_min_, C_last_) along with corresponding time points (t_max_, t_min_) were observed directly. Area under the concentration vs. time curve from time 0 to 12 h post-dose (AUC_0–12_) was estimated with the trapezoidal rule. The half-life (t_1/2_) was calculated as 0.693/λ_z_ where λ_z_ is the elimination constant derived by the terminal slope of the log concentration vs. time curve. Apparent oral clearance (CL/F) from plasma was calculated as dose divided by AUC_0–12_. Undetectable concentrations of etravirine were set at half the lower limit of quantification to calculate summary statistics. The IMPAACT protocol compared real time etravirine pharmacokinetic data with those from historical controls reported in the literature. Median trough concentrations of etravirine in non-pregnant adult studies were 0.25 mcg/mL. The 50th percentile AUC_0–12_ in non-pregnant adult studies was 4.2 mcg*h/mL and the P1026s study's minimal exposure cut-off was the 10th percentile AUC_0–12_ of 2.5 mcg*h/mL to ensure treatment efficacy (Boffito et al., 2007). The half maximal effective concentration (EC50) of etravirine is 0.004 mcg/mL against wild-type HIV-1 (Product information, 2014).

### Statistical analyses

The primary objective of this study was to compare pharmacokinetic parameters in pregnant HIV-infected women with paired postpartum data. Paired two-tailed Wilcoxon signed rank tests (α = 0.05) were employed to compare pharmacokinetic parameters in pregnant and non-pregnant women. A two-tailed Mann–Whitney *U*-test was used to compare pharmacokinetic parameters between subjects with and subjects without concomitant use of a ritonavir-boosted protease inhibitor with a two-sided *p* < 0.05. Statistical analysis was performed with Stata Statistical Software 2013, graphs were constructed using SlideWrite Plus 7.01 and tables were constructed using Microsoft Excel 2010.

## Results

### Subject characteristics

Fifteen pregnant women taking etravirine 200 mg twice daily enrolled in this study. Data were available for five subjects in the 2nd trimester. In the 3rd trimester, two women were lost to follow up and ten additional women enrolled in the study (*n* = 13). Postpartum data were available for one subject that began in the 2nd trimester and seven subjects that began in the 3rd trimester; five subjects were lost to follow up after the 2nd trimester (*n* = 8). Paired data were available for eight women that underwent sampling in the 3rd trimester and postpartum. Cord/delivery samples data were available for six mother-infant pairs. Subject demographic and clinical characteristics are summarized in Table 1. Among the 13 women available to follow-up, there were 13 live born infants for which data were available and the median (range) birth weight was 3205 g (2620–3800).

**Table 1:** **Subject demographics**.

**RACE/ETHNICITY**	
Black non-hispanic	7 (46.7%)
Hispanic	7 (46.7%)
Other	1 (6.7%)
Age at 3rd trimester (years)	25.6 (19.5–42.66)
Weight at 3rd trimester visit (kg)	76.6 (48–101.5)
Gestational age at 3rd trimester visit (weeks)	33.7 (30.3–36.7)
Weeks after delivery at postpartum PK visit	5.9 (3–8.4)
**OTHER ANTIRETROVIRALS**	
Raltegravir	9 (60%)
Darunavir, ritonavir	6 (40%)
Truvada (emtricitabine, tenofovir)	4 (27%)
Kaletra (lopinavir, ritonavir)	3 (20%)
Combivir (lamivudine, zidovudine)	2 (13%)
Maraviroc	2 (13%)
Duration of etravirine therapy at 3rd trimester visit (weeks)	12.7 (4.6–91.6)
**HIV-1 RNA AT DELIVERY**	
<50 copies/mL	9 (75%)
<400 copies/mL	10 (83%)
CD4 at delivery	420 (107–610)
Infant gestational age at birth (weeks)	38.6 (36–41.7)
Infant weight at birth (g)	3205 (2620–3800)
Infant length at birth (cm)	50 (45–52)
**INFECTION STATUS**	
Negative based on best available data	13 (100%)

*N = 15 for pregnant HIV-infected women and n = 13 for infants. Data are represented as n (%) or median (range)*.

### Etravirine pharmacokinetics

**Figure 1:**
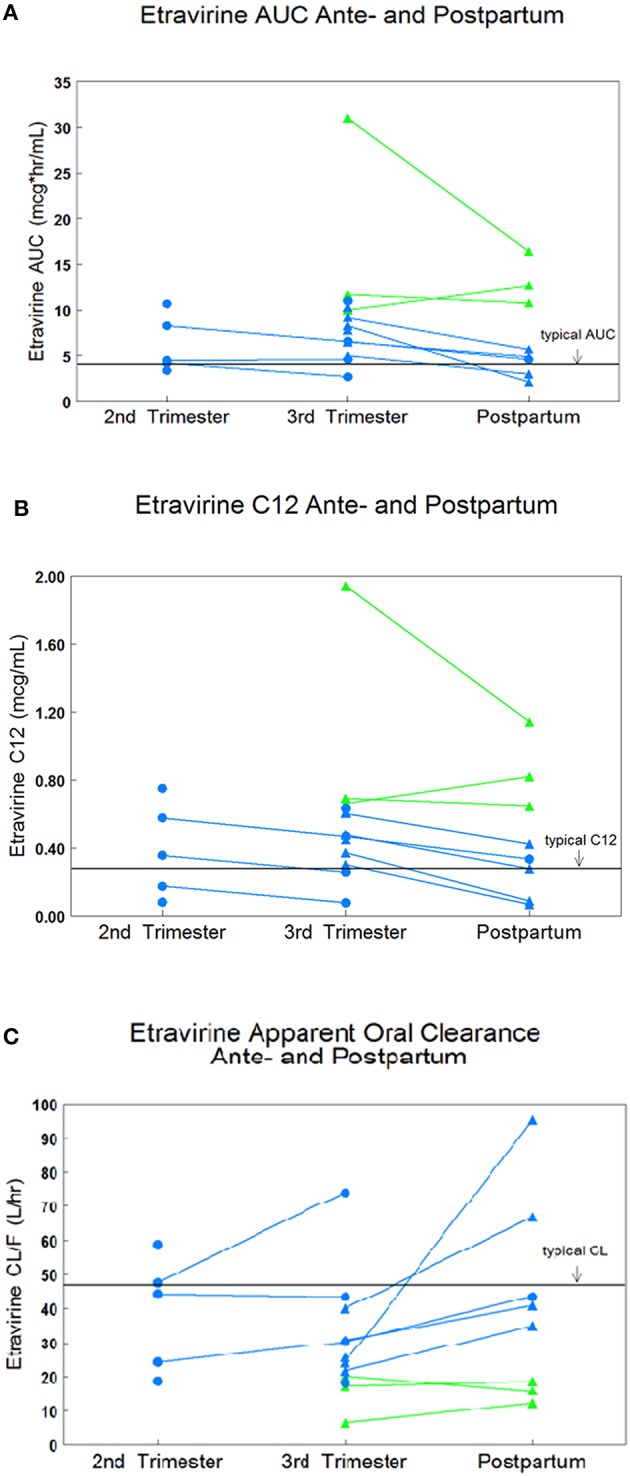
**IMPAACT subjects are represented in blue and PANNA subjects are represented in green**. **(A)** Paired AUC_0–12_ data in 2nd trimester, 3rd trimester, and postpartum. **(B)** Paired C_12_ data in 2nd trimester, 3rd trimester, and postpartum. **(C)** Paired CL/F data in 2nd trimester, 3rd trimester, and postpartum.

**Figure 2:**
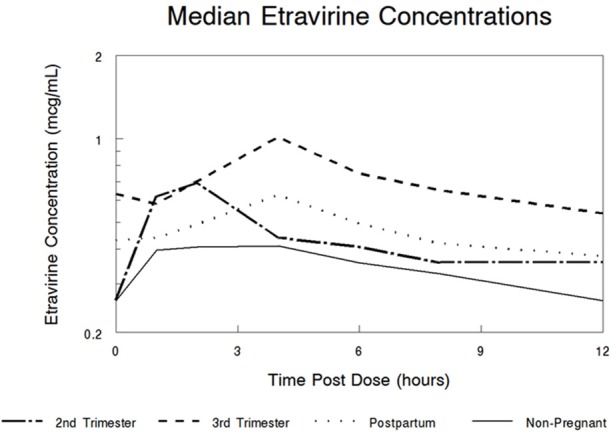
**Median etravirine (ETV) concentration-time curves during the 2nd trimester, 3rd trimester, and postpartum on 200 mg twice-daily dosing**. Non-pregnant adult reference line is seen in solid line (Boffito et al., 2007).

**Figure 3:**
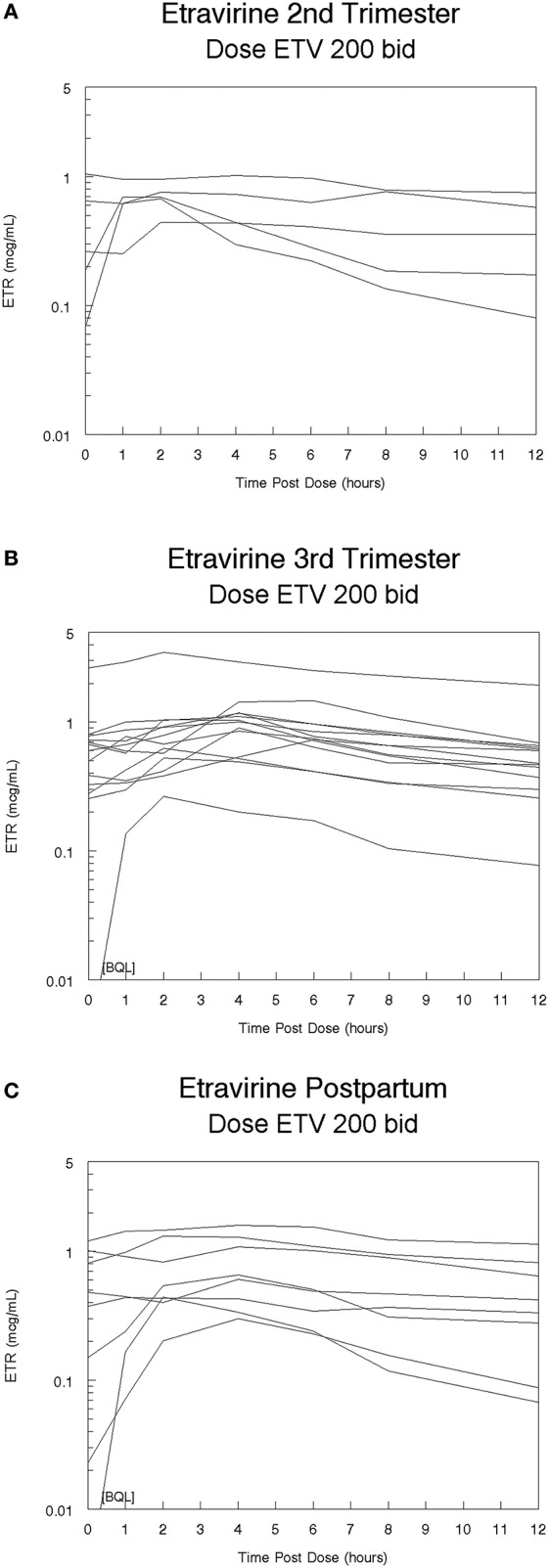
**(A)** Etravirine (ETV) concentration-time curves during the 2nd trimester following 200 mg twice-daily dosing, **(B)** Etravirine (ETV) concentration-time curves during the 3rd trimester following 200 mg twice-daily dosing, **(C)** Etravirine (ETV) concentration-time curves postpartum following 200 mg twice-daily dosing.

High inter-subject variability in etravirine pharmacokinetics was observed. Etravirine CL/F was significantly lower in the 3rd trimester of pregnancy compared to paired postpartum data with a median CL/F reduction of 52% (*p* = 0.0251). Median (range) etravirine CL/F during the 2nd trimester, 3rd trimester, and postpartum periods were 44 L/h (19–59), 24 L/h (6–74), and 38 L/h (12–95), respectively, (Figure 1). Etravirine maximum and 12-h concentrations were significantly higher in 3rd trimester of pregnancy compared to paired postpartum data with median elevations of 39 and 36%, respectively (*p* = 0.036, *p* = 0.036). During the 2nd trimester, 3rd trimester and postpartum periods, median (range) etravirine C_max_ were 0.70 mcg/mL (0.44–1.05), 1.01 mcg/mL (0.26–3.47), and 0.63 mcg/mL (0.30–1.60) and C_12_ were 0.36 mcg/mL (0.08–0.75), 0.48 mcg/mL (0.08–1.94), and 0.38 mcg/mL (0.07–1.14; Figures 2, 3).

A trend in increased etravirine AUC_0–12_ in the third trimester compared to postpartum was seen however, the difference did not reach statistical significance (*p* = 0.0684). Median (range) etravirine AUC_0–12_ during the 2nd trimester, 3rd trimester, and postpartum periods were 4.5 mcg*h/mL (3.4–10.7), 8.3 mcg*h/mL (2.7–31.0), and 5.3 mcg*h/mL (2.1–16.4), respectively. The frequency of subjects meeting the estimated AUC_0–12_ 10th percentile (2.5 mcg*h/mL) from non-pregnant historical controls was 5/5 (100%) subjects in the 2nd trimester, 13/13 (100%) subjects in the 3rd trimester, and 7/8 (88%) subjects postpartum. The frequency of subjects with AUC_0–12_ above the 90th percentile (5.86 mcg*h/mL) estimated from non-pregnant historical controls was 2/5 (40%) subjects in the 2nd trimester, 10/13 (77%) subjects in the 3rd trimester, and 3/8 (38%) subjects postpartum. The frequency of subjects meeting the 10th or 90th percentile across time points was not statistically significant (*p* = 0.310, *p* = 0.139). All other pharmacokinetic parameters were similar between 2nd trimester, 3rd trimester, and postpartum (Table 2). Pre-dose concentrations below the lower limit of detection were seen in one subject in the 3rd trimester and in another subject postpartum. These values suggest non-adherence but were still included in analysis.

**Table 2:** **Etravirine pharmacokinetic parameters**.

**Parameter**	**2nd Trimester *n* = 5**	**3rd Trimester *n* = 13**	**Postpartum *n* = 8**	**Geometric mean 3rd/Post Ratio**	**Historical Control**
AUC_0–12_ (mcg*h/mL)	4.5 (3.4–10.7)	8.3 (2.7–31.0)	5.3 (2.1–16.4)	1.34	6.03 (Schöller-Gyüre et al., 2009)
C_0_ (mcg/mL)	0.26 (0.07–1.05)	0.60 (<0.005–2.64)	0.43 (<0.005–1.21)	2.03	0.28 (0.10–0.85) (Boffito et al., 2007)
C_max_ (mcg/mL)	0.70 (0.44–1.05)	1.01 (0.26–3.47)^*^	0.63 (0.30–1.60)	1.34	0.44 (0.20–1.50) (Boffito et al., 2007)
T_max_ (h)	2 (0–8)	4 (2–6)	4 (1–4)	1.10	4 (1–6) (Schöller-Gyüre et al., 2009)
C_12_ (mcg/mL)	0.36 (0.08–0.75)	0.48 (0.08–1.94)^*^	0.38 (0.07–1.14)	1.41	0.47 (Schöller-Gyüre et al., 2009)
C_min_ (mcg/mL)	0.25 (0.07–0.75)	0.45 (<0.005–1.94)	0.38 (<0.005–1.14)	1.94	0.24 (0.09–0.73) (Boffito et al., 2007)
CL/F (L/h)	44 (19–59)	24 (6–74)^*^	38 (12–95)	0.75	^†^

*AUC_0-12_, area under the concentration vs. time curve from 0 to 12 h; C_0_, pre-dose concentration; C_max_, maximum concentration; T_max_, time at which maximum concentration occurred; C_12_, 12 h post-dose concentration; C_min_, minimum observed concentration; T_min_, time at which minimum concentration occurred; CL/F, oral clearance. Data are represented as median (range)*.

* p < 0.05 for 3rd trimester vs. postpartum values using Wilcoxon signed-rank test. For other pharmacokinetic parameters in the 3rd trimester and postpartum, n = 13 and 8, respectively. Historical data AUC_0–12_ mean was 4.2 mcg*h/mL among non-pregnant adults (Boffito et al., 2007) and 6.03 mcg*h/mL among non-pregnant women (Schöller-Gyüre et al., 2009).

† *Describes data which was absent in referenced study*.

Third trimester exposure was compared between subjects with and subjects without concomitant use of a protease inhibitor (PI) boosted by ritonavir (*n* = 9, *n* = 4). Median (range) AUC_0–12_-values were 5.6 mcg*h/mL (2.7–11) without a boosted PI and 9.2 mcg*h/mL (5–31) with a boosted PI (*p* = 0.182). Median (range) CL/F-values were 36.9 L/h (18.2–74.1) without a boosted PI and 21.7 L/h (6.5–40) with a boosted PI (*p* = 0.162). Median (range) C_min_-values were 0.30 mcg/mL (0–0.632) without a boosted PI and 0.57 mcg/mL (0.28–1.94) with a boosted PI (*p* = 0.165). Etravirine exposure tended to increase and CL/F to decrease with protease inhibitor use, however, these differences were not statistically significant due to small subject populations.

Maternal plasma samples at delivery and umbilical cord blood samples were collected from six subjects (Table 3). Median (range) etravirine concentrations were 0.22 mcg/mL (0.05–2.89) in umbilical cord blood and 0.38 mcg/mL (0.11–0.68) in maternal plasma. The median (range) ratio of cord blood to maternal plasma concentration at delivery was 0.52 (range: 0.19–4.25). The median (range) time between administration of the last antenatal etravirine dose and delivery was 10.3 h (5.1–29.6).

**Table 3:** **Placental passage (***n*** = 7)**.

**Parameter**	**Median (range)**
Cord blood concentration (mcg/mL)	0.22 (0.05–2.89)
Maternal plasma concentration (mcg/mL)	0.38 (0.11–0.68)
Cord blood/maternal plasma ratio	0.52 (0.19–4.25)

*Cord blood and maternal plasma concentrations were collected after cord clamping*.

### Maternal and infant outcomes

In the course of this study, two women reported grade 3 adverse events: fever and elevated glucose concentrations. In the 3rd trimester, 10/13 (77%) of subjects had an HIV-1 RNA ≤ 50 copies/mL, and one subject had a viral load over 400 copies/mL. At delivery, 9/12 (75%) maternal subjects had an HIV-1 RNA ≤ 50 copies/mL and two subjects had a viral load over 400 copies/mL (Table 1). At postpartum pharmacokinetic sampling, 6/7 (86%) maternal subjects achieved an HIV-1 RNA ≤ 50 copies/mL. HIV-RNA testing results were not available within the study window from 1 subject in the third trimester and 1 subject postpartum.

Data were available for 13 study infants. Infants were born at a median of 38.6 weeks of gestation (range: 36–41.7) with a median birth weight of 3205 g (range: 2620–3800). Three infants had congenital anomalies: skin tag on ear, bilateral double 5th toe, and patent foramen ovale with right to left shunt and right ventricular hypertrophy. Four infants had Grade 3 or 4 laboratory abnormalities: glucose (*n* = 1), potassium (*n* = 1), and absolute neutrophil count (*n* = 3). The patent foramen ovale, ventricular hypertrophy, and abnormal glucose were in the same infant. Patent foramen ovale is an abnormality related to the physiological changes at birth therefore, the maternal subject's etravirine exposure was excluded from the following “abnormality” grouping. Among mothers of infants with anomalies, two women used etravirine prior to pregnancy, one woman began etravirine use in the first trimester, and one women began use in the second trimester of pregnancy. In the 2nd trimester, mothers of infants with anomalies had an average etravirine AUC_0–12_ of 9.5 mcg*h/mL (*n* = 2) compared to an AUC_0–12_ of 4.0 mcg*h/mL (*n* = 3) for mothers of infants without anomalies. In the 3rd trimester, mothers of infants with anomalies had a median (range) etravirine AUC_0–12_ of 7.45 mcg*h/mL (6.5–11.0, *n* = 4) compared to an AUC_0–12_ of 9.6 mcg*h/mL (2.7–31.0, *n* = 9) for mothers of infants without anomalies. Cord blood to maternal plasma ratios were 0.48 (*n* = 2) in infants with anomalies compared to 0.52 (0.2–4.3, *n* = 5) in infants without anomalies. All (100%) infants were HIV negative based on best available data.

## Discussion

Maintaining therapeutic antiretroviral exposure during pregnancy is crucial for maternal health outcomes and reducing perinatal transmission of HIV. An etravirine AUC_0–12_ below the 10th percentile (2.5 mcg*h/mL) of non-pregnant adults was designated the pharmacokinetic target and 100% of pregnant subjects and 89% of postpartum subjects met this target (Boffito et al., 2007). Furthermore, C_12_ in all subjects and gestational time points exceeded the *in vitro* etravirine EC_50_ for wild type HIV-1 (0.004 mcg/mL) by one to three orders of magnitude (Product information, 2014). These findings suggest that standard etravirine dosage for non-pregnant adults, 200 mg twice-daily, provides sufficient exposure in pregnancy. Ten (77%) subjects achieved undetectable HIV-1 RNA levels (≤ 50 copies/mL) at delivery. Among subjects that were not lost to follow up, seven (88%) subjects had undetectable HIV-1 RNA levels at postpartum pharmacokinetic sampling.

This study found that etravirine apparent oral clearance was significantly reduced by 52% in the 3rd trimester of pregnancy compared to paired postpartum data. The maximum and 12-h plasma concentrations of etravirine were significantly increased by 39 and 36% in the 3rd trimester compared to paired postpartum data. Though not significant, a trend was seen for elevated AUC_0–12_ in the 3rd trimester compared to postpartum. It is likely this finding did not reach statistical significance due to the small numbers of paired data (*n* = 9). During the third trimester, 11 (85%) subjects had an AUC_0–12_ above the estimated 90th percentile (5.86 mcg*h/mL) of historical non-pregnant adult controls (Boffito et al., 2007). Etravirine exposure appears to be increased in the 3rd trimester of pregnancy.

Other assessments of etravirine pharmacokinetics in pregnant HIV-infected women include two case studies, a case series of data from four women and a presentation of data from a phase IIIb pharmacokinetic study of 15 pregnant women. In the case series of four pregnant patients, observed medians (ranges) in the 3rd trimester were: AUC_0–12_ of 4.5 mcg*h/mL (3.0–8.9), C_0_ of 0.41 mcg/mL (0.15–0.90), T_max_ of 3 h, C_max_ of 0.69 mcg/mL (0.45–1.15), and CL/F of 44.3 L/h (22.6–65.8; Izurieta et al., 2011). In comparison to this study, the case series observed lower AUC_0–12_ and C_max_-values, comparable T_max_-values, and higher C_0_ and CL/F-values during pregnancy; all parameters fell within one standard deviation of this study's findings. The case series participants were generally a decade older than this study's subjects. In one case study, a 29 week multigestational pregnancy had a C_0_ of 0.90 mcg/mL, T_max_ of 6 h, and a C_max_ of 1.21 mcg/mL (Furco et al., 2009). It is difficult to make a direct comparison of multi-gestational pregnancy to single-gestational pregnancy. Data from the phase IIIb trial of 15 pregnant women was comparable to our findings with mean AUC_0–12_-values of 6.6 ± 2.8, 6.8 ± 1.5, and 5.0 ± 2.5 mcg*h/mL in the 2nd trimester, 3rd trimester, and postpartum, respectively. Subjects were older than in this study with a median (range) of 26 years (20–34) and 73% of subjects were black (Ramgopal et al., 2016). The gender, race and clinical experience (GRACE) trial has shown a direct relationship between age and etravirine exposure but no significant differences across race in non-pregnant adults (Kakuda et al., 2012). Phase III trials and a pharmacogenetic-based population pharmacokinetic study have identified high intersubject variability in etravirine exposure in non-pregnant adults with 16% of variability attributed to genetics (Kakuda et al., 2010; Lubomirov et al., 2013; Ramgopal et al., 2016).

A case study, a case series of data from 4 women and the pharmacokinetic study of 15 pregnant women also reported data on the transplacental passage of etravirine. The median (range) cord blood to maternal plasma ratio of etravirine in this study was 0.52 (0.19–4.25, *n* = 6) and higher than in 3 previous studies: 0.32 (0.19–0.63), 0.51, and 0.33 (Izurieta et al., 2011; Calcagno et al., 2013; Ramgopal et al., 2016).

The DUET-I and DUET-II clinical trials of etravirine were administered with boosted darunavir (DRV/r) and several subsequent studies have evaluated etravirine use with DRV/r in the non-pregnant adult population (Schöller-Gyüre et al., 2007; Kakuda et al., 2008, 2010; Katlama et al., 2010; Lubomirov et al., 2013). Concomitant DRV/r significantly increases etravirine apparent oral clearance by 40% in the non-pregnant adult population (Schöller-Gyüre et al., 2007; Kakuda et al., 2010; Lubomirov et al., 2013). Two studies have also analyzed concomitant use of etravirine with boosted lopinavir (LPV/r; Lubomirov et al., 2013; Schöller-Gyüre et al., 2013). Concomitant LPV/r also increases etravirine apparent oral clearance in the non-pregnant adult population but not significantly (Lubomirov et al., 2013; Kakuda et al., 2010; Schöller-Gyüre et al., 2007). For this reason, we compared 3rd trimester etravirine concentrations between patients with (*n* = 9) and without (*n* = 4) use of boosted protease inhibitors (6 with DRV/r, 3 with LPV/r). No significant differences were seen in 3rd trimester etravirine AUC_0–12_, CL/F and C_min_ but our small sample size limits the power of this analysis. These findings oppose previous study findings in non-pregnant adults. Due to our study's small subject population and high intersubject variability, more data are needed to elucidate the effect of DRV/r or LPV/r on etravirine pharmacokinetics in the pregnant population. It is possible that the influence of pregnancy on increasing etravirine exposure may outweigh DRV/r or LPV/r effects on decreased etravirine exposure.

Increased etravirine exposure during the 3rd trimester of pregnancy is a unique finding among antiretroviral drugs. This finding is likely due to the unique metabolism of etravirine by CYP3A4, CYP2C9, and CYP2C19 (Product information, 2014). Many antiretroviral drugs are metabolized by CYP3A4, and show evidence of lowered exposure in pregnancy (Panel on Treatment of HIV-Infected Pregnant Women and Prevention of Perinatal Transmission, 2014). In contrast, CYP2C9 and CYP2C19 contributes to the metabolism of few antiretrovirals. Metabolite identification experiments identified CYP2C19 as the primary metabolizer of etravirine yielding two major metabolites. The same study found CYP3A4 and CYP2C9 form several minor metabolites (Yanakakis and Bumpus, 2012). During pregnancy, enzyme expression of CYP2C19 is suppressed while CYP3A4 and CYP2C9 are induced (Anderson, 2005; Ke et al., 2014). Our findings of decreased etravirine apparent oral clearance in the 3rd trimester of pregnancy is consistent with reduction in CYP2C19 expression in pregnancy resulting in higher etravirine plasma concentrations. Both CYP2C19 and CYP2C9 have well-described genetic variations associated with impaired drug metabolism which accounts for some interpatient variability (Lubomirov et al., 2013). Subjects of this study were not genotyped.

Increases in total drug exposure typically raise concerns about toxicity. Furthermore, etravirine is 99.9% protein bound, primarily to albumin and alpha 1-acid glycoprotein (Product information, 2014). In pregnancy, increases in several hormones compete for protein binding while an increase in volume has a dilutional effect on plasma proteins (Mirochnick and Capparelli, 2004). Very small losses in the number of protein binding sites is also a concern as this can significantly impact free-drug concentrations and the risk of toxicity. While we did not measure free concentration of etravirine in plasma, etravirine was generally well-tolerated in our subjects with only two reports of grade 3 or 4 adverse events. A dose-ranging study with etravirine 400, 800, and 1200 mg twice-daily found no dose relationship to adverse event frequency or severity (Montaner et al., 2008). Combined with a mild adverse event profile, primarily rash and nausea with rare hypersensitivity reactions, multiple dose-ranging trials have stated that no dose adjustment is necessary in elevated etravirine exposure (Kakuda et al., 2007, 2008; Montaner et al., 2008). We identified moderate placental transfer of etravirine to the fetus with a 0.52 cord blood to maternal ratio, so elevated maternal etravirine plasma concentrations could also lead to high fetal concentrations (Panel on Treatment of HIV-Infected Pregnant Women and Prevention of Perinatal Transmission, 2014). High fetal concentrations may be beneficial in prophylaxis against HIV-infection or may cause toxicities in the infant. Thirteen (100%) of this study's infants were HIV negative based on best available data. This study identified three infants (23%) with congenital anomalies and four (31%) with grade 3 or 4 laboratory abnormalities. Aside from case studies, no published literature describes etravirine exposure in infants. While elevated etravirine concentrations do not require dose adjustments for maternal health, it remains unclear if standard adult dosing of etravirine in pregnancy will have significant clinical impact on the fetus. The antiretroviral pregnancy registry interim report described 1/54 birth defects in 54 first trimester etravirine exposures between January 1st 1989 through January 31st 2015. This is below the background rate of congenital anomalies (The Antiretroviral Pregnancy Registry Steering Committee, 2015). Three of the five women with infants displaying congenital or laboratory anomalies in this study were exposed to etravirine in the first trimester of pregnancy.

Several study limitations must be considered. Opportunistic recruitment of pregnant women already receiving etravirine as part of their clinical care with 2 week stability on the combination ART regimen excludes early signs of toxicity, intolerance, virologic failure, and other reasons for medication discontinuation. This exclusion introduces bias toward pregnant women that tolerated and responded to etravirine therapy. Medication adherence and dosing relative to meals were self-reported. Since food impacts etravirine absorption, unidentified poor adherence, or recall bias may have added greater variability to our findings (Lubomirov et al., 2013). Postpartum sampling occurred between 3 and 12 weeks following delivery. During the postpartum period, physiological changes occur rapidly and our postpartum pharmacokinetic data has high variability as compared to other time points. A majority of study participants were black or Hispanic in their twenties. Studies have shown a relationship between age and etravirine exposure but no significant differences across race (Schöller-Gyüre et al., 2009; Kakuda et al., 2010; Lubomirov et al., 2013). Subjects enrolled at various stages of pregnancy and duration of etravirine use ranged from 4.6 to 91.6 weeks at the time of third trimester sampling. However, phase III trials found that etravirine reaches steady-state exposure by 4 weeks in non-pregnant adults (Kakuda et al., 2010).

In summary, the 200 mg twice-daily dosing of etravirine recommended for non-pregnant adults achieves elevated exposure in the 3rd trimester of pregnancy. Previous studies have shown no relationship between etravirine exposure and adverse event incidence or severity suggesting no dose adjustment is necessary for maternal health. However, it remains unclear how elevated maternal etravirine concentrations in the 3rd trimester will impact infant health. Close monitoring of mother and infant outcomes is warranted.

## Author contributions

All co-authors reviewed, revised for content, and approved this article. Additionally, AC, JM, AS, GT, CH, CF, and MV substantially contributed to data acquisition. NM, SS, BB, AC, JW, EC, AS, ES, NC, MM, and DB substantially contributed to conception and design of study, data analysis and data interpretation.

## Funding

Overall support for the International Maternal Pediatric Adolescent AIDS Clinical Trials Group (IMPAACT) was provided by the National Institute of Allergy and Infectious Diseases (NIAID) of the National Institutes of Health (NIH) under Award Numbers UM1AI068632 (IMPAACT LOC), UM1AI068616 (IMPAACT SDMC) and UM1AI106716 (IMPAACT LC), with co-funding from the Eunice Kennedy Shriver National Institute of Child Health and Human Development (NICHD) and the National Institute of Mental Health (NIMH). The content is solely the responsibility of the authors and does not necessarily represent the official views of the NIH. Overall support for the “Pharmacokinetics of newly developed antiretroviral agents in HIV-infected pregnant women (PANNA)” network is financially supported by the “European AIDS Treatment Network (NEAT)” under the European Commission, DG Research, 6th Framework program, contract LSHP-CT-2006-037570 and the “Pediatric European Network for Treatment of AIDS (PENTA)” under the European Commission, DG Research, 5th Framework program, contract QLK2-CT-2000-00150 and 6th Framework program, contract LSHP-CT-2006-018865, with co-funding from Merck Sharp & Dohme Corp, Bristol Myers Squibb, Janssen Research and ViiV Healthcare.

### Conflict of interest statement

The authors declare that the research was conducted in the absence of any commercial or financial relationships that could be construed as a potential conflict of interest.
